# Cell cycle synchronisation using thiazolidinediones affects cellular glucose metabolism and enhances the therapeutic effect of 2-deoxyglucose in colon cancer

**DOI:** 10.1038/s41598-020-61661-4

**Published:** 2020-03-13

**Authors:** Joon-Kee Yoon, Hye Eun Byeon, Seung Ah Ko, Bok-Nam Park, Young-Sil An, Ho-Young Lee, Youn Woo Lee, Su Jin Lee

**Affiliations:** 1 Department of Nuclear Medicine and Molecular Imaging, Ajou University School of Medicine, Suwon, Republic of Korea; 2 Institute of Medical Science, Ajou University School of Medicine, Suwon, Republic of Korea; 3 Department of Nuclear Medicine, Seoul National University Bundang Hospital, Seongnam, Republic of Korea

**Keywords:** Cancer, Molecular biology, Molecular medicine, Oncology

## Abstract

The effect of cell cycle synchronisation on glucose metabolism in cancer cells is not known. We assessed how cell cycle synchronisation by thiazolidinediones (TZDs) can affect glucose uptake by cancer cells and investigated the anti-cancer effect of combination therapy with TZDs and 2-deoxy-glucose (2-DG) in colon cancer cells and in mouse xenograft models. Troglitazone (58.1 ± 2.0 vs 48.6 ± 1.3%, *p* = 0.002) or pioglitazone (82.9 ± 1.9 vs 61.6 ± 3.4%, *p* < 0.001) induced cell cycle arrest in SW480 cells at G1 phase. Western blot analysis showed the degradation of cyclin D1 and CDK4, and an increase in the expression levels of p21 and p27 after TZDs treatment. Withdrawal of troglitazone treatment induced significant increase in cellular ^3^H-DG uptake (141.5% ± 12.9% of controls) and membrane GLUT1 expression levels (146.3% of controls) by 24 h; 1 mM 2-DG treatment alone decreased cell survival by 5.8% as compared with the controls.; however, combination therapy enhanced the anti-tumour effects to 34.6% or 20.3% as compared with control cells. *In vivo*, each combination treatment group showed significant anti-tumour effects unlike the 2-DG alone group. Cell cycle synchronisation using TZDs induced cellular glucose uptake, which significantly enhanced the therapeutic effect of 2-DG in colon cancer.

## Introduction

The reprogramming of energy metabolism has recently been recognised as a hallmark of cancer^1^. Understanding metabolic differences between normal cells and cancer cells can provide opportunities for both cancer imaging and therapy. Imaging glycolytic shifts in tumours using ^18^F-fluorodeoxyglucose (FDG) positron emission tomography (PET), which has been widely used in clinical oncology, is a notable example. To treat cancers by targeting tumour metabolism, 2-deoxyglucose (2-DG) inhibits glycolysis, depletes intracellular adenosine triphosphate, and induces autophagy in cancer cells^2^. However, as high concentrations of 2-DG are needed to inhibit the glycolytic metabolism of cancer cells^2,3^, approaches that can enhance the efficacy of 2-DG treatment are crucial.

Until now, a lot of agents that can modulate cell cycle in cancer cells have been used or are under development for cancer therapy^4^. However, few studies have examined the effect of cell cycle synchronisation on glucose metabolism in cancer cells. We have previously reported that cell cycle arrest at G1 by T-type calcium channel blocker, mibefradil, induced a subsequent increase in cell population in S phase after drug removal, and increased glucose uptake s in breast cancer cells, which enhanced the therapeutic effects of 2-DG^5^.

Peroxisome proliferator activated receptor-γ (PPARγ) is widely expressed in various tumours and cell lines, thus this receptor has become a target for developing new anticancer drugs that can take advantage of the antiproliferative effects mediated through PPAR. Thiazolidinediones (TZDs), such as troglitazone, pioglitazone, ciglitazone, and rosiglitazone are well-known PPARγ ligands. TZDs have been reported to exert anti-tumour effects, such as apoptosis induction, differentiation, and growth (proliferation) arrest via PPARγ-dependent or -independent mechanisms^6^. Several studies have shown that the expression of cyclin D1 was downregulated by TZDs in colon cancer, breast cancer, and prostate cancer as part of the mechanism for causing G1 cell-cycle arrest and growth inhibition of cancer cells^7–10^.

In this study, we assessed how cell cycle synchronisation using TZDs can affect glucose metabolism in colon cancer cells. We further investigated whether the anticancer effect of 2-DG can be augmented when used in combination therapy with TZDs *in vitro* and *in vivo*.

## Results

### Effect of TZDs on cell cycle progression

**Figure 1:**
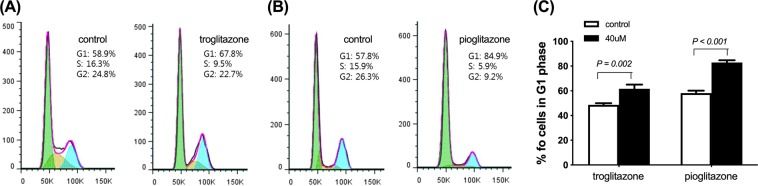
Effect of troglitazone and pioglitazone on cell cycle. Representative cell cycle analysis performed by flow cytometry treated with troglitazone (**A**) or pioglitazone (**B**) for 24 hours. (**C**) The percentage of cells in G1 phase of cell cycle after troglitazone or pioglitazone treatment. All results are mean ± SD of triplicate samples expressed as percentage relative to controls obtained from single representative experiment of 3 separate experiments.

We examined the effect of TZDs on cell cycle progression in SW480 cells. Treatment with 40 μM troglitazone or pioglitazone for 24 h successfully induced G1 arrest while there was decrease in cell population in S phase (Fig. 1A,B). The proportion of SW480 cells in G1 phase following treatment with troglitazone (58.1% ± 2.0% vs 48.6% ± 1.3%, *p* = 0.002) or pioglitazone (82.9% ± 1.9% vs 61.6% ± 3.4%, *p* < 0.001) was significantly higher than that of the controls (Fig. 1C).

**Figure 2:**
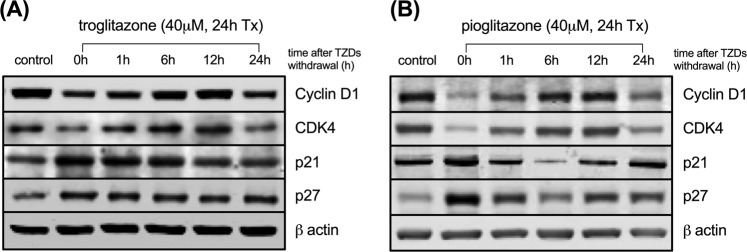
Changes of cell cycle-associated protein expression after troglitazone (**A**) or pioglitazone (**B**) withdrawal. Results are obtained from a single representative experiment of three separate experiments.

To further investigate the molecules affected by TZDs, we examined the expression levels of several G1 or S phase-associated proteins, such as cyclins, cyclin dependent kinases (CDKs), p21, and p27 under similar conditions. While cyclin D1 and CDK4 protein levels were downregulated by treatment with 40 μM troglitazone or pioglitazone for 24 h, the expression levels of CDK inhibitors, p21 and p27, were increased. The withdrawal of TZDs normalised these protein levels at 6 h to 12 h post-treatment (Fig. 2A,B). The results show that TDZs can induce the degradation of cyclin D1 and CDK4, and the expression of p21 and p27, which is consistent with their inhibition of G1 to S-phase progression in colon cancer cells.

### Change of glucose metabolism after cell cycle synchronisation by TZDs

**Figure 3:**
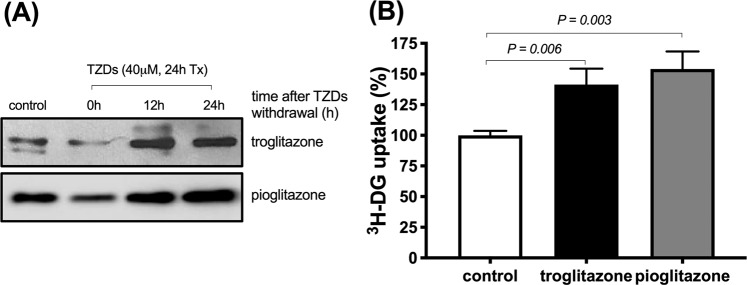
Changes of membrane GLUT1 expression (**A**) and accumulated ^3^H-DG uptake (**B**) after troglitazone or pioglitazone treatment. Results are obtained from single representative experiment of 3 separate experiments.

Under similar conditions as followed in the above cell cycle experiment, we measured the change of membranous glucose transporter 1 (GLUT1) expression at 0, 12, and 24 h and 24 h-accumulated ^3^H-DG uptake after removing TZDs. Membranous GLUT1 expression decreased immediately after troglitazone or pioglitazone treatment. However, the expression of GLUT1 increased at 12 h and 24 h after stopping treatment (Fig. 3A). Accumulated ^3^H-DG uptake increased significantly for both troglitazone (141.5% ± 12.9% of controls) and pioglitazone (154.0% ± 14.3% of controls, Fig. 3B). These results indicate that ^3^H-DG uptake increases through the enhanced glucose metabolism by means of cell cycle modification.

### Treatment with TZDs and 2-DG enhanced the anti-tumour effect of 2-DG *in vitro* and *in vivo*

**Figure 4:**
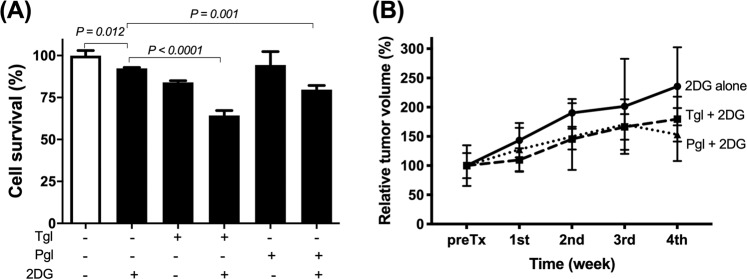
Therapeutic effects of the combination therapy with thiazolidinediones and 2-DG in SW480 cells (**A**) and mouse models (**B**). Results are obtained from single representative experiment of two separate experiments. Tgl, troglitazone; Pgl, pioglitazone.

Sulforhodamine-B (SRB) assay demonstrated that 1 mM 2-DG treatment alone could slightly suppress cell survival by 5.8% as compared to that of untreated control colon cancer cells. Troglitazone treatment alone also suppressed cell survival significantly by 16.7% (*p* = 0.001). Unlike single therapies, the combination therapy (troglitazone treatment followed by 2-DG treatment) showed synergistic effect by killing 34.6% of cells (Fig. 4A). For pioglitazone, a single therapy failed to show significant cytotoxic effect (5.7% decrease as compared with controls); however, combination therapy with 2-DG showed significant anti-cancer effect (20.3%, Fig. 4A).

In the xenograft mouse model, combination therapy groups showed enhanced anti-cancer effects compared to that observed in the 2-DG treatment group at the completion of therapy (Fig. 4B). There was no significant difference of tumour volumes between the groups before treatment. After completion of therapy, relative tumour volumes were 235.6% ± 66.8% for 2-DG alone, 179.5% ± 38.5% for troglitazone with 2-DG group, and 153.2% ± 45.2% for pioglitazone with 2-DG group, respectively.

**Figure 5:**
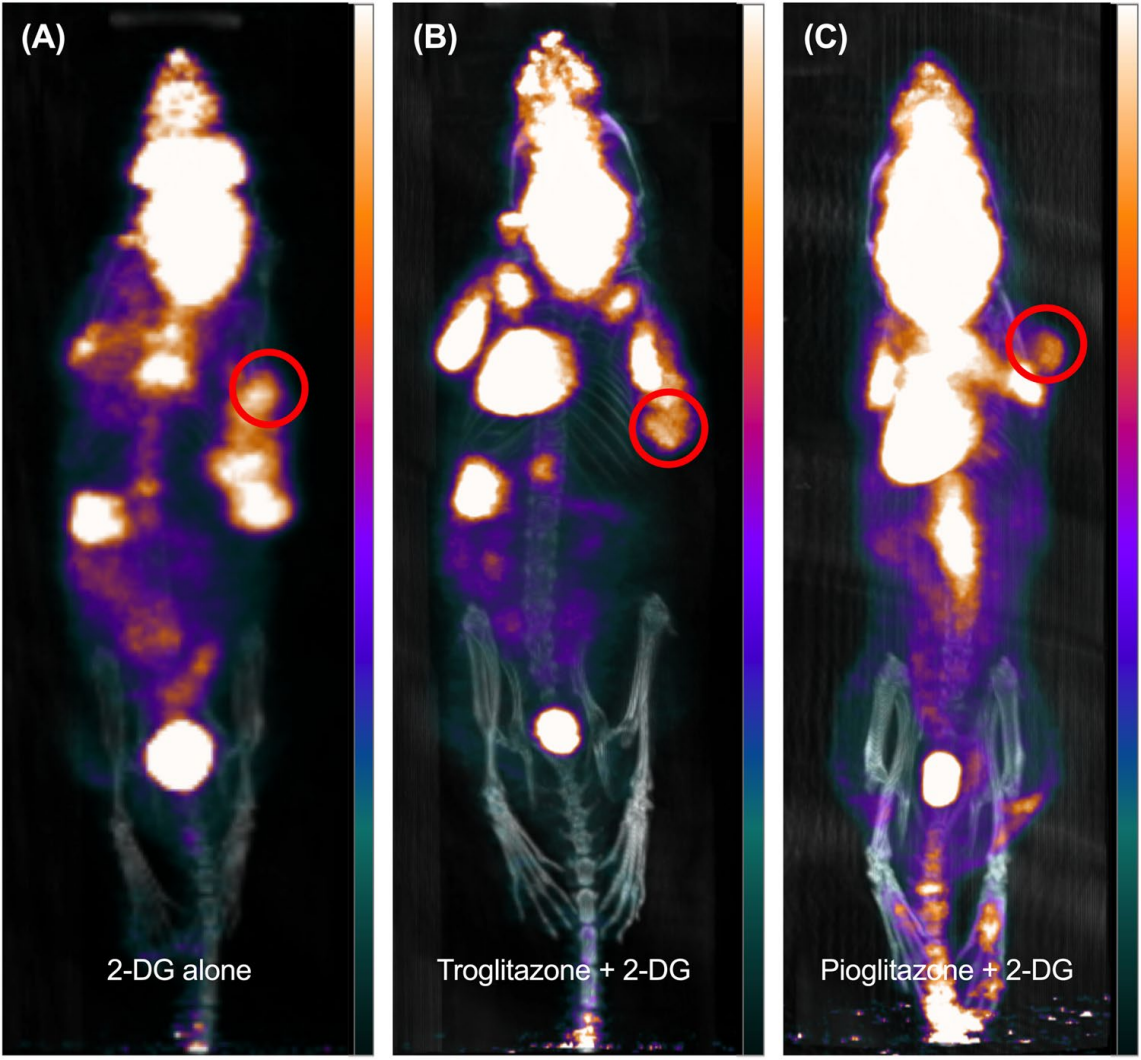
Representative micro-PET/CT images in mouse model. Maximum intensity projection images of 2DG alone (**A**), troglitazone with 2DG group (**B**) and pioglitazone with 2DG group (**C**). Red circle indicates tumour.

Representative PET/CT images of each group are shown in Fig. 5. The mean SUV of 2-DG alone, troglitazone with 2-DG group, and pioglitazone with 2-DG group were 0.22, 0.17, and 0.09 g/ml, respectively. In addition, MTV of 2-DG alone, troglitazone with 2-DG group, and pioglitazone with 2-DG group were 0.68, 0.22, and 0.05 cm^3^, respectively.

## Discussion

This study demonstrated that combining 2-DG with either troglitazone or pioglitazone can increase the therapeutic efficacy of 2-DG. Cell cycle synchronisation using troglitazone or pioglitazone, and then subsequent G1-S transition following drug removal induced a significant increase in glucose uptake, which led to an enhanced anti-tumour effect of 2-DG *in vitro* and *in vivo*. As far as we know, this study is the first investigation about the therapeutic effects of combination therapy using cell cycle synchronisation and 2-DG in mouse tumour models. Until now, several preclinical and clinical studies have been performed to evaluate the efficacy of 2-DG treatment in combination with chemotherapy or radiation therapy in lung cancer, osteosarcoma, and human cerebral glioma^11–14^. Although, results from those studies lacked consensus, 2-DG showed significant improvement in achieving partial or complete response when used prior to radiotherapy or following chemotherapeutic drugs, such as adriamycin, paclitaxel or etoposide.

New combination therapies using cell-cycle agents have already been studied^15^. These are CDK inhibitors, Cdc25 phosphatase inhibitors, cell cycle checkpoint inhibitors, and mitotic inhibitors that cause cell cycle arrest followed by induction of apoptosis. The mechanism of our combination therapy differs from those agents. We focused on the modulation of glucose metabolism mediated by cell cycle synchronisation and the resultant enhancement in the therapeutic efficacy of 2-DG. Initially, we optimised the correlation between alterations in cell cycle, especially G1-S phase transition, and ^3^H-DG uptake using double thymidine block in SW480 colon cancer cells. Double thymidine block is a well-known method for G1 arrest *in vitro*. We induced G1 arrest with double thymidine block and then observed the changes in cell cycle and glucose uptake in colon cancer cells. Significant increase in glucose uptake was accompanied by normalisation of cell cycle within 24 h after G1 arrest (Supplementary Data 1). Therefore, G1-S transition can induce significant increase in glucose uptake in cancer cells.

In our previous study^5^, we used a T-type calcium channel inhibitor, mibefradil, to induce G1 cell cycle arrest in breast cancer cells. We observed that G1 arrest and subsequent entry to S-phase by the removal of drug significantly increased the cytotoxic effects of 2-DG *in vitro*, either as a single agent or in combination with paclitaxel. This enhanced anti-tumour effect arose from the increased ^3^H-DG uptake *via* increased glycolysis in cancer cells. Contrary to the above-mentioned cell cycle agents, mibefradil did not increase apoptosis.

In the present study, we selected TDZs as an inducer for G1 arrest in SW480 colon cancer cells. Troglitazone and pioglitazone are antidiabetic drugs, although troglitazone was withdrawn from the antidiabetic market due to hepatotoxicity. Recently, the strategy for reprofiling drug with troglitazone has been tried; unsaturated troglitazone derivatives exhibit a higher efficiency for cancer cells and a lower toxicity towards hepatocytes than troglitazone^16^. In this study, both troglitazone and pioglitazone induced cell cycle arrest at G1 phase, which increased 2-DG uptake in cancer cells and augmented its therapeutic efficacy. These findings are broadly consistent with previous observations by Lapela *et al*. in non-Hodgkin lymphoma^17^; the percentage of cells in S phase was closely related to glucose utilisation. Our study suggested that TDZs can be used as cell-cycle agents in cancer treatment.

*In vitro* study, the combination therapy using troglitazone with 2-DG showed better anti-tumour effect than the therapy using pioglitazone with 2-DG (p = 0.002). However, no significant difference of therapeutic effects between the two combination therapies was observed in the xenograft mouse models (p = 0.996). Although further clinical trials may be needed to prove the therapeutic effect of combination therapy, pioglitazone may be preferable to troglitazone because pioglitazone is currently used as an antidiabetic drug in the clinical practice.

Since cell cycle is tightly regulated through a complex network of positive and negative regulatory molecules, the induction of cyclin D1 and CDK4 expression with concomitant downregulation of CDK inhibitors, p21 and p27, is necessary for transition through G1 phase. As expected, cyclin D1 and CDK4 were downregulated while p21 and p27 were upregulated after treatment with troglitazone or pioglitazone.

*In vivo* study, we compared the therapeutic effects between combination therapy group and 2-DG alone group because tumours in controls (no treatment) grow rapidly, the mice could not survive until the completion of 4-week treatment. In addition, the purpose of this study was to evaluate the enhancement of therapeutic efficacy of 2-DG; thus, we compared the therapeutic effects between the two groups.

One limitation of this study is that we performed *in vitro* and *in vivo* studies using only one colon cancer cell line. Further studies with other malignant tumours are needed to confirm the anti-cancer effects of this combination therapy. In conclusion, cell cycle synchronisation using TZDs induced the cellular glucose uptake, which significantly enhanced the therapeutic effect of 2-DG *in vitro* and *in vivo* colon cancer models.

## Methods

### Cell culture

SW480 cancer cell line was obtained from the Korean Cell Line Bank (KCLB No.10228). Cells were cultured in RPMI-1640 medium (HyClone) supplemented with 10% foetal bovine serum (HyClone) and 1% penicillin/streptomycin (Gibco), and were maintained under full humidity at 37 °C and 5% CO_2_.

### Cell cycle analysis

Cell cycle analysis was performed as described previously in our study^5^. Colon cancer cells were seeded into 6-well plates and cultured overnight. For the treatment group, 40 μM troglitazone or pioglitazone (Sigma-Aldrich) was added, and cells were incubated for 24 h. Cells were then collected by trypsinisation at 0 h and 24 h after removing troglitazone or pioglitazone, rinsed twice in 5 ml of 0.1% bovine serum albumin/ phosphate-buffered saline (PBS), and fixed with 3 ml cold 70% ethanol. Samples were then stored at −20 °C until needed. On the day of analysis, samples were transferred to flow cytometry tubes and washed twice with PBS. After removing the supernatant by centrifugation, the cell pellet was resuspended in 3 ml PBS containing 0.1% Triton X-100, 10 μg/ml propidium iodide and 100 μg/ml RNase A, and then incubated at room temperature for 30 min. Cell cycle analysis was then done by flow cytometry (BD Bioscience) and FlowJo software version 7.6 (www.flowjo.com/solutions/flowjo).

### Measurement of ^3^H-2-deoxyglucose uptake

Cells were seeded into 12-well plates and cultured overnight. After incubation with 40 μM troglitazone for 24 h, cells were washed with warm PBS. Then cells were incubated in low glucose culture media containing 18.5 kBq ^3^H-2-DG for up to 24 h. After washing with cold PBS twice, cells were lysed with 0.1 N NaOH. Radioactivity was measured using a liquid scintillation counter (Tri-Carb 2100TR). The cellular uptake of treated groups was presented as the percentage of cellular uptake of controls after normalisation to protein content that was calculated using Pierce-660 (Thermo Fisher Scientific) and a microplate reader (VMax; Molecular Devices). Experiments were performed in triplicates.

### Immunoblotting for cell cycle-associated protein and glucose transporter 1

For immunoblotting of cell cycle-associated proteins, cells were washed twice with PBS and lysed in radioimmunoprecipitation assay buffer containing protease inhibitor cocktail (Bioprince). For immunoblotting of glucose transporter 1 (GLUT1), cells were washed twice with PBS and membrane proteins were isolated using plasma membrane protein isolation kit (Invent Biotechnologies). Protein concentrations were measured using protein assay kit (Thermo Fisher Scientific). Thirty to fifty micrograms of each sample were electrophoresed on sodium dodecylsulphate polyacrylamide gels and transferred to nitrocellulose membranes. The membranes were blocked with 5% skim milk in Tris-buffered saline/ Tween-20 (TBST) for 1 h. The membranes were incubated with primary antibodies for 1 h at room temperature or overnight at 4 °C. They were then washed three times with TBST for 5 min and incubated with IRDye secondary antibody (LI-COR Biosciences) for 1 h. The membranes were visualised using an Odyssey infrared imaging system (LI-COR Biosciences). Antibodies for cyclin D1, CDK4, p21, and p27 were purchased from Cell Signalling Technology. Antibodies for β-actin and GLUT1 were purchased from Santa Cruz Biotechnology, and Abcam, respectively.

### Sulforhodamine-B (SRB) assay

**Figure 6:**
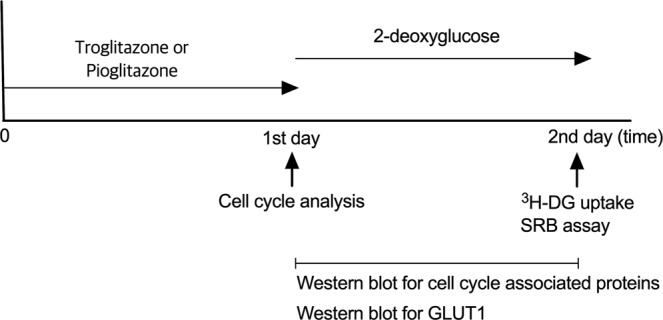
Experimental protocol of *in vitro* models.

Cytotoxic effects were evaluated by SRB assay. Cells were divided into 4 groups; control, troglitazone or pioglitazone treatment, 2-DG treatment, and combination therapy (troglitazone or pioglitazone treatment followed by 2-DG treatment) groups. Cells were seeded into 96-well plates and cultured overnight. For troglitazone or pioglitazone treatment group and combination therapy group, cells were first incubated with 40 μM troglitazone or pioglitazone for 24 h. The cells were then incubated in fresh media (for control and troglitazone or pioglitazone treatment group) or fresh media containing 1.0 mM 2-DG (for 2-DG treatment group and combination therapy group) for a further 24 h. For the SRB assay, cells were fixed in 3.3% (w/v) trichloroacetic acid (Sigma-Aldrich) at 4 °C for 1 h. After removing fixative solution from the plates, cells were incubated with 100 μl of 0.057% (w/v) SRB (Sigma-Aldrich) at room temperature for 10 min. Cells were then rinsed four times with 1% (v/v) acetic acid and dried. Stained cells were dissolved in 100 μl 10 mM Tris base (pH 10.5) for 10 min with shaking. Finally, absorbance at 490 nm was measured on a microplate reader. *In vitro* experimental protocols are summarised as Fig. 6. Experiments were performed in triplicates.

### Xenograft tumour model and treatment

The animal study was approved by the Institutional Animal Care and Use Committee (IACUC) at Ajou University School of Medicine (IACUC No. 2017-0044) and all methods were performed in accordance with the relevant guidelines and regulations. Six-week-old female Balb/c nude mice (OrientBio) were maintained under specific pathogen-free conditions. To induce xenografts, 1×10^6^ of SW480 cells in phenol red-free Matrigel (BD Biosciences) were injected subcutaneously into the dorsal region of the right thigh of each mouse. Treatment was initiated about 2 weeks after inoculation when the tumour size reached 5 mm to 10 mm in diameter. Mice were randomly assigned to 3 groups; 2-DG treatment group, troglitazone with 2-DG treatment group, and pioglitazone with 2-DG treatment group, respectively. Troglitazone or pioglitazone was dissolved in Solutol HS-15 (9% in PBS, Sigma-Aldrich), which is a nontoxic solvent and is composed of polyglycol mono- and di- esters of 12-hydroxystearic acid and 30% free polyethylene glycol. All drugs and vehicles were injected intraperitoneally. 2-DG (1000 mg/kg bw) was administered once daily, 5 days a week, for 4 weeks^11^; while troglitazone, pioglitazone (60 mg/kg bw) or vehicle was administered once daily, 3 days a week, for 4 weeks. *In vivo* experimental protocols are summarised as Supplementary Data 2.

Optimum therapeutic dose of TZDs (60 mg/kg) was determined through pilot studies in xenograft models. Thirty mg/kg of troglitazone for mice is similar to the therapeutic dose for humans (200 mg)^18^ if corrected for interspecies dose scaling factors^19^. However, our preliminary experiments using 30 mg/kg (3 times/week for 4 weeks) did not show significant antitumor effects, thus the dose was increased to 60 mg/kg in this experiment.

The inhibitory effect was assessed by comparing the volumes of tumour xenografts, which were calculated using the following formula: TV = length × width^2^/2 (TV: estimated tumour volume [mm^3^]).

### Animal PET/ CT acquisition and imaging analysis

In total, 15 mice (n = 5 for each group) were used for FDG PET/CT imaging after the therapy completion. Mice were fasted at least 6 h before intravenous administration of ^18^F-FDG. Mice were injected with 18.5 MBq of ^18^F-FDG *via* tail vein and PET/CT images were acquired after 60 min after injection (Nano PET/CT, Bioscan). During the image acquisition, the mice were anaesthetised (5%) and sustained (1%~2%) with isoflurane in a 7:3 mixture of N_2_/O_2_.

Analysis of the PET/CT images was performed using PMOD version 3.9 (PMOD Technologies, https://www.pmod.com). Polygonal regions of interest were manually drawn with care to include whole tumour volumes. The radiotracer uptake in each ROI was estimated as the standardised uptake value (SUV), which was calculated as decay-corrected activity per millilitre of tissue divided by injected radiotracer activity per body mass ([kBq/mL]/[kBq/g]). Metabolic tumour volume (MTV) was measured with fixed absolute threshold and SUV 0.02 was used as threshold.

### Statistical analysis

Results are expressed as the mean ± standard deviation. The percentage of cell cycle phase, ^3^H-DG cellular uptake, cell viability, and the tumour volumes were compared between groups using the Student’s t-test, with 0.05 as a cut-off for p-value.

## Supplementary information

Supplementary information.

## Data Availability

The datasets used and/or analysed during the current study are available from the corresponding author on reasonable request.
