# Supplementary material for: Cell cycle synchronisation using thiazolidinediones affects cellular glucose metabolism and enhances the therapeutic effect of 2-deoxyglucose in colon cancer
Source: Sci Rep. 2020 Mar 13;10:4713. doi: 10.1038/s41598-020-61661-4 (PMC7070066; doi:10.1038/s41598-020-61661-4)
Supplement: Supplementary file 1 — Supplementary information. [file 41598_2020_61661_MOESM1_ESM.pdf]

Cell cycle synchronisation using thiazolidinediones affects cellular glucose metabolism and enhances the therapeutic effect of 2-deoxyglucose in colon cancer

Joon-Kee Yoon[1*], Hye Eun Byeon[2*], Seung Ah Ko[1], Bok-Nam Park[1], Young-Sil An[1], Ho-Young Lee[3], Youn Woo Lee[3], and Su Jin Lee[1]

Cell synchronization using a double thymidine block

For cell synchronization, SW480 cells at 40% confluence were washed twice with PBS and first incubated with 2 mM thymidine (Sigma-Aldrich) for 19h. After the first thymidine block, cells were incubated in fresh medium. After 9h, cells were re-treated with 2 mM thymidine for 16h. This process synchronized SW480 cells at the G1/S border. Cells were cultured for different times as indicated in the experiment and harvested.

Cell cycle synchronization using a double thymidine block induces substantial increase of ^{3}H-DG uptake. (supplementary data 1)

G1 arrest was successfully induced after cell cycle synchronization using a double thymidine block (Fig 1a). Cells were replaced with fresh media, cell cycle analysis using FACS was performed at 1h, 6h, 12h, and 24h, respectively. Cell cycle started to normalize.

For evaluation of cellular glucose metabolism after G1 arrest, cells were washed with warm PBS, and then incubated in low glucose culture media containing 18.5 kBq ^{3}H-2-DG for up to 24 h. Immediately after G1 arrest, cells showed slight decrease of ^{3}H-2-DG

uptake, however glucose uptake was substantially increased up to 24 h (2798.4 ± 591.7 % of controls; Fig 1b).

Supplementary Fig 1a

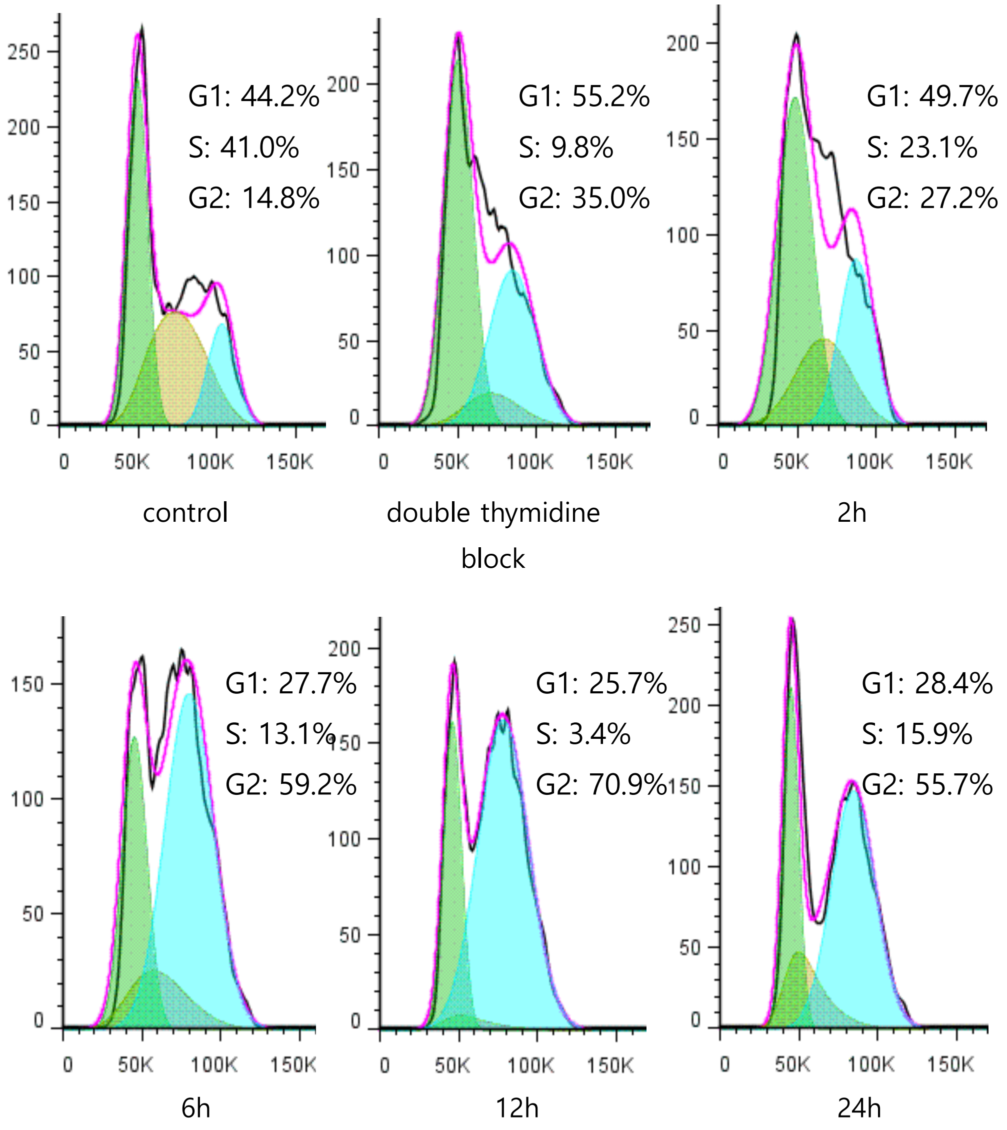

Supplementary Fig 1b

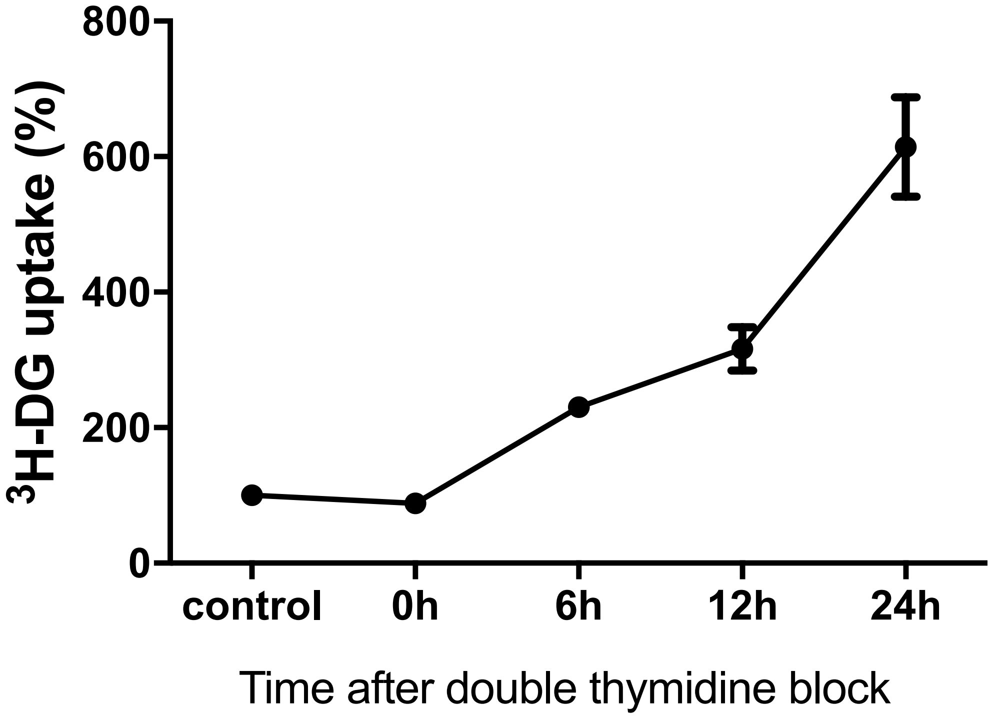

Treatment protocols of xenografts model (supplementary data 2)

weekly schedule

	1	2	3	4	5 (days)
Group 1	2DG	2DG	2DG	2DG	2DG
Group 2	Tgl + 2DG	2DG	Tgl + 2DG	2DG	Tgl + 2DG
Group 3	Pgl + 2DG	2DG	Pgl + 2DG	2DG	Pgl + 2DG

Tgl, troglitazone; Pgl, pioglitazone

Treatment was performed for 4 weeks.
